# Deoxycytidine kinase inactivation enhances gemcitabine resistance and sensitizes mitochondrial metabolism interference in pancreatic cancer

**DOI:** 10.1038/s41419-024-06531-x

**Published:** 2024-02-12

**Authors:** Suman Dash, Takeshi Ueda, Akiyoshi Komuro, Masahiko Honda, Ryoichi Sugisawa, Hitoshi Okada

**Affiliations:** 1 Department of Biochemistry, Kindai University Faculty of Medicine, Osakasayama, Osaka 589-8511 Japan; 2 Graduate School of Medical Sciences, Kindai University Faculty of Medicine, Osakasayama, Osaka 589-8511 Japan; 3 Anti-aging Center, Kindai University, Higashi-Osaka, Osaka 577-8502 Japan

**Keywords:** Cancer metabolism, Cancer therapeutic resistance

## Abstract

Pancreatic ductal adenocarcinoma (PDAC) is considered one of the most lethal forms of cancer. Although in the last decade, an increase in 5-year patient survival has been observed, the mortality rate remains high. As a first-line treatment for PDAC, gemcitabine alone or in combination (gemcitabine plus paclitaxel) has been used; however, drug resistance to this regimen is a growing issue. In our previous study, we reported MYC/glutamine dependency as a therapeutic target in gemcitabine-resistant PDAC secondary to deoxycytidine kinase (DCK) inactivation. Moreover, enrichment of oxidative phosphorylation (OXPHOS)-associated genes was a common property shared by PDAC cell lines, and patient clinical samples coupled with low DCK expression was also demonstrated, which implicates DCK in cancer metabolism. In this article, we reveal that the expression of most genes encoding mitochondrial complexes is remarkably upregulated in PDAC patients with low DCK expression. The DCK-knockout (DCK KO) CFPAC-1 PDAC cell line model reiterated this observation. Particularly, OXPHOS was functionally enhanced in DCK KO cells as shown by a higher oxygen consumption rate and mitochondrial ATP production. Electron microscopic observations revealed abnormal mitochondrial morphology in DCK KO cells. Furthermore, *DCK* inactivation exhibited reactive oxygen species (ROS) reduction accompanied with ROS-scavenging gene activation, such as *SOD1* and *SOD2*. *SOD2* inhibition in DCK KO cells clearly induced cell growth suppression. In combination with increased anti-apoptotic gene *BCL2* expression in DCK KO cells, we finally reveal that venetoclax and a mitochondrial complex I inhibitor are therapeutically efficacious for DCK-inactivated CFPAC-1 cells in in vitro and xenograft models. Hence, our work provides insight into inhibition of mitochondrial metabolism as a novel therapeutic approach to overcome *DCK* inactivation-mediated gemcitabine resistance in PDAC patient treatment.

## Introduction

Pancreatic ductal adenocarcinoma (PDAC) is a fatal form of cancer in which the 5-year survival rate remained at 12% [1]. The main drawbacks of PDAC are delayed diagnosis, rapid metastasis and development of chemotherapy resistance [2]. Currently, surgical resection and chemotherapy are commonly considered as the primary treatment options for PDAC. However, surgery is indicated in only 15–20% of patients at the time of diagnosis owing to the presence of local and distant metastases [3]. Therefore, clinical treatment depends on systemic chemotherapy for most patients. This highlights the urgent need to understand the molecular basis of treatment resistance in PDAC [4].

Gemcitabine, a deoxycytidine nucleoside analog, was initially demonstrated to improve PDAC survival in 1997 and continues to be used as a fundamental metabolite in PDAC management [5, 6]. Although patients initially respond well to gemcitabine therapy, gemcitabine resistance eventually develops secondary to reduced cellular uptake, increased efflux rate, intracellular transformation, reactivation of pivotal developmental pathways such as Hedgehog (Hh), Wnt, and Notch in gemcitabine-resistant cells [6, 7]. The rate-limiting enzyme responsible for gemcitabine activation, deoxycytidine kinase (DCK), is frequently inactivated in PDAC cells with acquired gemcitabine resistance [8, 9]. Furthermore, recent genome-wide screen also confirmed the significance of *DCK* inactivation in gemcitabine resistance in PDAC cells [10]. Moreover, PDAC patients with decreased *DCK* expression exhibited shorter overall survival during gemcitabine-based treatment [11]. Hence, characterizing DCK-inactivated and downregulated cells is crucial for the improvement of pancreatic cancer treatment outcomes.

Tumor cells require an efficient energy source for biochemical precursor synthesis in maintaining an active cell proliferation to sustain tumor growth. It is now widely recognized that ATP can be simultaneously generated through both glycolysis and OXPHOS pathways in cancer cells, including PDAC [12, 13]. PDAC cells show diverse metabolic heterogeneity and are subtyped as “glycolytic,” “lipogenic,” and “slow proliferating” according to their preference for bioenergetics and metabolic inhibitor responsiveness [14]. Recent studies have demonstrated that PDAC, similar to other malignancies, undergoes substantial metabolic reprogramming, which enhances tumor development and resistance to therapy [15, 16]. Previously, it has been shown that pancreatic cancer with stem cell features and subpopulation of dormant cells depend on OXPHOS to ensure their survival [17, 18]. Additionally, OXPHOS activity affects mitochondrial morphology in pancreatic cancer [19, 20]. Regarding gemcitabine sensitivity in PDAC cell lines, it has been noted that cells with a high OXPHOS activity are less sensitive to gemcitabine [21]. Therefore, understanding the correlation between DCK ablation-mediated gemcitabine resistance and OXPHOS modulation is crucial to improve patient survival.

In our previous study, we elucidated on the upregulation MYC/glutamine dependence and OXPHOS-related genes in DCK-deficient gemcitabine-resistant cells [10]. In this study, further characterization of the DCK-deficient PDAC cells showed that these cells have increased mitochondrial gene set expression associated with a higher OXPHOS activity for energy production. Additionally, comparable gene expression patterns were identified in patients with PDAC with a low DCK expression. Also, the DCK-deficient PDAC cells exhibited a higher dependence on ROS scavenging and anti-apoptotic pathways compared to DCK-proficient PDAC cells. Consequently, gemcitabine-resistant cells induced by *DCK* inactivation can be effectively targeted by utilizing a mitochondrial complex I inhibitor in vitro cell culture and mouse xenograft models. Collectively, these findings indicate a potential therapeutic approach to tackle gemcitabine-resistant PDAC cells induced by *DCK* inactivation.

## Methods and materials

### Cell lines

The human PDAC cell line, CFPAC-1, was acquired from the American Type Culture Collection, and cell line authentication was confirmed using Promega GenePrint 10 system. The CFPAC-1 cell line was transduced with oligos and corresponded to the sgRNA sequence (Supplementary Table 1) of sgDCK#10 and control sgNT1 described in our previous article and named as DCK#10 and NT1 cell [10]. CFPAC-1, DCK#10 (DCK knockout CFPAC-1 cell), NT1 (CFPAC-1 cell with the non-target gRNA insert), HA-DCK (DCK#10 with stable expression of HA-DCK [10]), HA-DCK-KD (DCK#10 with stable expression of HA-DCK bearing the kinase-dead mutation [10]) and EV (DCK#10 with stable expression of empty vector [10]) cells were maintained in Iscove’s Modified Dulbecco’s Medium (IMDM; FUJIFILM Wako Pure Chemical Corporation [FUJIFILM-Wako]), HPAF-II cells were maintained in Eagle’s minimum essential medium (FUJIFILM-Wako). All cell culture media were supplemented with 10% heat-inactivated FBS (Gibco #10270–106) and 1% penicillin–streptomycin (10,000 U/mL; Thermo Fisher Scientific, #15140–122). Cells were cultured in a humidified atmosphere containing 5% CO_2_ at 37 °C and maintained in culture for less than 20 passages. Mycoplasma testing was conducted using MycoBlue mycoplasma detector kit (Vazyme).

### Gene set enrichment analysis (GSEA)

Gene expression data for GSEA were obtained from DRX370437-DRX370440 in the DNA Data Bank of Japan for CFPAC-1 and DCK-deficient CFPAC-1 (DCK#10) cell lines and the Pan-Cancer Atlas (The Cancer Genome Atlas [TCGA]) [22] for patients with PDAC. GSEA [23] was conducted to compare the following groups: CFPAC-1 versus DCK#10 and PDAC patients with a high DCK expression (*n* = 11, Z-score > 1.25) versus those with a low DCK expression (*n* = 12, Z-score < −1.25). Using the following gene sets, analyses were performed: gene ontology cellular component, biological process and molecular function (GOCC, GOBP, and GOMF, respectively), human phenotype ontology from MSigDB, and mitochondrial respiratory chain complexes from HGNC. The GSEA software was used to calculate the normalized enrichment score. A false discovery rate *q*-value of <0.25 was considered significant.

### Western blotting

Western blotting was performed following the protocol described in our previous publication [10]. Proteins related to mitochondrial complexes were probed using Total OXPHOS Rodent WB Antibody Cocktail (abcam, ab #110413, 1:1000). BCL2 protein was detected with anti-BCL2 antibody (Proeintech, #12789-1-AP, 1:2000). DCK protein was identified using anti-DCK antibody produced in rabbits (GeneTex, #GTX102800, 1:1000). As a loading control, anti-β-actin antibody (Santa Cruz, #sc-47778, 1:1000) was used.

### Real-time quantitative reverse transcription polymerase chain reaction (RT-qPCR)

Using the Monarch Total RNA Miniprep Kit (NEB #T2010), total RNA was extracted from cells. For the tissue samples, DCK#10 and NT1 cell-derived xenograft tissues were collected, and total RNA was extracted using the TRIzol reagent (Thermo Fisher #15596026) according to the manufacturer’s protocol. The iScript cDNA Synthesis Kit (Bio-Rad #1708840) was used to synthesize the cDNA. RT-qPCR was conducted using the Luna universal qPCR master mix (NEB #M3003L), with primers specific for the target genes of interest. The *GUSB* gene was used as an internal control. The primer sequences are presented in Supplementary Table 2. PCR was performed as follows: initial incubation step was performed at 95 °C for 30 s, followed by 40 cycles of 95 °C for 5 s and 60 °C for 30 s. Data were collected using the StepOnePlus Real-Time PCR System (Applied Biosystems). The specific PCR amplification was verified using a dissociation curve with a single peak and via electrophoresis of the PCR products.

### Ectopic DCK expression

To achieve ectopic overexpression of DCK, DCK was subcloned into the lentiviral expression vector CSII-CMV-MCS-IRES2-Bsd (RIKEN BRC #RDB04385) at the NheI/XhoI site, following the procedure described previously [10]. Subsequently, CFPAC-1 and HPAF-II cells transduced with the lentivirus were selected in IMDM and EMEM media containing 10 μg/mL of blasticidin S for 6 days. Ectopic expression was confirmed by western blotting.

### Measurement of energy metabolism

To measure mitochondrial function, Seahorse XF Cell Mito Stress Test Kit from Agilent technologies (AT [AT #103015-100]) was used according to the manufacturer’s instruction. DCK#10 and NT1, HA-DCK, HA-DCK-KD, along with EV cells, were seeded at 1 × 10^4^ cells/well of a standard XFe96 microplate and incubated at 37 °C overnight using a regular culture medium. The next day, the culture medium was replaced with a seahorse XF DMEM medium at pH 7.4 (AT #103575-100) supplemented with 10 mmol/L glucose (AT #103577-100), 1 mmol/L pyruvate (AT #103578-100), and 2 mmol/L l-glutamine (AT #103579-100), and the plate was pre-incubated for 1 h at 37 °C in a non-CO_2_ incubator. Under basal conditions, the oxygen consumption rate (OCR) was measured and then a series of different reagents were introduced through sequential injections for further analysis. To measure mitochondrial function, the final concentration of oligomycin (1.5 μmol/L), carbonyl cyanide-p-trifluoromethoxyphenylhydrazone (FCCP, 1 μmol/L), and rotenone plus antimycin A (0.5 μmol/L) was selected according to our preliminary experiments. Seahorse XFp cell energy phenotype test kit (AT #103275-100) was used for measuring cellular metabolic potential for both DCK#10 and NT1 cells. An equal number of cells and a similar assay medium was used to acquire the energetic profile of the cells. Extracellular acidification rate (ECAR) was measured using Seahorse XF Glycolysis Stress Kit (AT #103020-100). Prior to initiation of the sequential treatment, non-glycolytic acidification or background ECAR was measured. To assess the basal glycolysis level, 10 mmol/L glucose was injected and glycolytic capacity was measured by injecting 1 μmol/L of oligomycin. At the end of the experiment, glycolysis was discontinued using 50 mmol/L 2-DG. An equal number of cells and the same assay medium was utilized to obtain the energetic profile of the cells. The flux analyzer measurements were conducted in five replicates and were repeated at least three times.

### Mitochondrial DNA content measurement

Mitochondrial DNA (mtDNA) copy number was measured using the protocol described by Venegas and Halberg [24]. Briefly, DNA was extracted from the DCK#10 and NT1 cells using a QIAamp DNA mini kit (Qiagen #51304). The extracted DNA was diluted to 2 ng/μL using a DNA hydration solution (10 μmol/L tris-HCl, 1-mM ethylenediaminetetraacetic acid in nuclease free, deionized water, pH = 8.0). qPCR was performed through adding 1 μL of 5 μmol/L forward and reverse primers and 3 μL of the DNA template to 5 μL of the Luna Universal qPCR Master Mix (NEB #M3003L), for a total volume of 10 μL. PCR was conducted as follows: at two steps initial incubation at 50 °C for 2 min and 95°C for 20 s respectively, followed by 40 cycles of 95 °C for 15 s and annealing at Tm for 30 s, then final extension at 95°C for 15 s, and cooling reaction at 50 °C for 15 s. Primers targeting two genes, namely, the mitochondrial genes tRNA^Leu(UUR)^ gene and mtDNA 16 S rRNA gene, were used; furthermore, nuclear β-2-microglobulin (β2M) primers were used for qPCR in which nuclear β2M was used as the internal control. The primer sequences are listed in Supplementary Table 2. The quantity of the mtDNA in DCK#10 cells was divided by the quantity of the mtDNA in NT1 cells to determine the relative mtDNA copy number.

### Electron microscopy

At a density of 3 × 10^4^ cell per well with regular IMDM media, cells were seeded on collagen-coated four-chambered slide (IWAKI #5722-004). After 48 h, when the cells were in the exponential growth phase, they were first fixed in 2.5% glutaraldehyde for 24 h at 4 °C, then the cells were rinsed in 0.1-M phosphate buffer (pH = 7.4), and post-fixed in 1% osmium tetraoxide for 1 h at 4 °C. After rinsing with distilled water, the cells were progressively dehydrated in a graded ethanol series. Subsequently, they were immersed in QY-1 (n-Butyl glycidyl ether; NISSHIN EM, Japan) and then embedded in epoxy resin. The ultrathin sections (with 80-nm thickness) were prepared with UC7 microtome (LEICA) and stained using uranyl acetate and lead citrate. Finally, the cell ultrastructures were examined using transmission electron microscopy (HT7700, Hitachi, Japan). Utilizing ImageJ software, mitochondrial circularity and length were quantified according to the images of NT1 and DCK#10 cells magnified to 1000× according to the protocol previously described [25].

### Measurement of intracellular reactive oxygen species (ROS)

Intracellular ROS levels were measured using flow cytometry (FACS) using H2DCFDA (2´,7´-dichlorodihydrofluorescein diacetate, Sigma #D6883) staining. Cells were seeded into a 6-well plate at a density of 5 × 10^5^ cells per well. After 24 h, cells were detached using trypsin-ethylenediaminetetraacetic acid and suspended in IMDM media supplemented with 10% FBS. The cells were then centrifuged at 3000 rpm for 3 min and resuspended in phosphate-buffered saline (PBS) supplemented with 2 μmol/L H2DCFDA and 1% FBS. This suspension was incubated at 37 °C for 1 h in the dark. A positive control was included using 250 μmol/L H_2_O_2_, which was treated for 1 h at 37 °C prior to staining with H2DCFDA. ROS measurements were performed in triplicate, and the entire experiment was repeated at least three times.

### Small interfering RNA (siRNA) transfection and cell proliferation assay

DCK#10 and NT1 cells were seeded at 3 × 10^4^ cell/well in a 12-well plate with regular culture media to perform the siRNA transfection. After 24 h, cells were transfected with 10 nmol/L ON-TARGETplus Human SOD2 siRNA pool (siSOD2; Horizon Discovery # L-009784-00-0005) or 10 nmol/L scrambled control siRNA (siControl; Horizon Discovery #D-001810-10-05) using Lipofectamine RNAiMAX Transfection Reagent (Invitrogen #13778-030) according to the manufacturer’s instruction. Cells were trypsinized and harvested in regular culture medium and then seeded in a 96-well plate at a density of 5,000 cells/well with 200-µL media for proliferation assay at 72 h post-transfection. After another 72 h, 20 µL of Cell Count Reagent SF (WST- 8 [2-(2-methoxy-4-nitrophenyl)-3-(4-nitrophenyl)-5-(2,4-disulfophenyl)-2H-tetrazolium]; Nacalai Tesque, #07553–15) was added to each well and incubated for 2 h at 37°C. Thereafter, the absorbance was promptly measured at 450 nm using a microplate reader (iMark; Bio-Rad). The background readings were subtracted from each original reading.

### Measurement of mitochondrial superoxide

Mitochondrial superoxide levels were measured using MitoSOX Green Mitochondrial Superoxide indicator (MSG) (Invitrogen, #M36005), following the method previously described [26, 27]. NT1 and DCK#10 cells were transfected with scrambled control (siControl) and human SOD2 siRNA pool as described above. After 72 h, the cells were detached using trypsin-ethylenediaminetetraacetic acid and suspended in IMDM media supplemented with 10% FBS. Subsequently, cells were washed three times with HBSS-Ca^2+^ and Mg^2+^ ((HBSS), Gibco, #14025-092) and centrifuged at 400 × *g* for 3 min after each wash. MSG reagent was prepared as a 1 mM working solution by dissolving in 10 μL of N, N Dimethylformamide ((DMF), FUJIFILM-Wako #043-32361) according to the manufacturer’s instructions. This solution was then diluted in HBSS to create the final working solution. The cells were resuspended with 0.5 μmol/L MSG at 37 °C for 20 min in the dark and washed three times with HBSS followed by centrifugation at 400 × *g* for 3 min after each wash. The fluorescence of MitoSOX Green was detected using BD FACS Canto II flow cytometer.

### Cell viability assay

Using Cell Count Reagent SF (WST-8; Nacalai Tesque, #07553–15), cellular viability was assessed. Cells were seeded into 96-well plates at 5000 cells per well with 200 µL of media 24 h prior to treatment with IACS-010759 (Selleck-chem #S8731) and venetoclax [(ABT-199) (Selleck-chem #S8048)] at the indicated doses. After 72 h of treatment, 20 µL of WST-8 was added to each well and incubated for 2 h at 37 °C, and then the absorbance was immediately measured at 450 nm via a microplate reader (iMark; Bio-Rad). The background readings were subtracted from each original reading. The cellular viability assay was conducted in triplicates and repeated at least three times. Using the curves constructed through plotting cellular viability versus drug concentration, the IC50 values were calculated [28].

### Cell viability assay by crystal violet staining

Cells were seeded in a 96-well plate at 5000 cells per well 24 h prior to treatment with the indicated doses of IACS-010759. Cells were washed with PBS and fixed with 0.2% crystal violet (Sigma, # C6158; dissolved in 80% methanol [FUJIFILM-Wako #131-01826]) after 72 h of treatment, incubated for 20 min at room temperature, and then the cells were immersed in tap water and air-dried. Picture was taken using a digital camera (OLYMPUS).

### Xenograft and in vivo treatment

Male, 4-week-old BALB/cAJcl-nu/nu mice were purchased from CLEA Japan Inc. Mice were allowed to acclimatize for 6 days. DCK#10 and NT1 cells (1 × 10^6^) were injected subcutaneously into the right flanks of the mice using a 0.15-mL cell suspension in a 1:1 mixture of serum-free IMDM and Matrigel (Corning #CLS256234). When the tumor became visible 10 days after transplantation, mice were randomly divided into two experimental groups and treated with vehicle (0.5% methyl cellulose; viscosity: 4000 cP; Sigma # M0512) and IACS-010759 (Selleck-chem: #S8731) through oral gavage. The vehicle and 7.5 mg/kg per dose of IACS-010759 were orally administered once daily on a 5-days-on and 2-days-off regimen as previously described [29]. The tumor volume was measured every other day using the following formula: *V* = ½ (*L* × *W*^2^) where L represents the longest side of the tumor and W is the shortest. Mice underwent daily monitoring and their body weight was measured every other day. Mice were housed and maintained in a specific pathogen-free mouse facility at Kindai University Faculty of Medicine with controlled environmental conditions including temperature (20–24 °C), humidity (40–70%), and a 12-h light–dark cycle. Mice were provided unrestricted access to standard laboratory chow and water. All experimental procedures were performed in accordance with institutional guidelines and approved protocols from the Institutional Animal Care and Use Committee.

### Statistical analysis

Results are expressed as mean ± standard deviation. Using an unpaired two-tailed Student’s *t*-test, differences between the two groups were analyzed. Multiple groups were compared using one-way analysis of variance. Statistical significance was set at *P* < 0.05.

## Results

### DCK inactivation and lower DCK expression levels correlate with higher mitochondrial functions

We recently reported that DCK deficiency was the primary mechanism involved in gemcitabine resistance in human PDCA cells through the CRISPR genome-wide knockout library screening [10]. RNA sequencing analysis from DCK-deficient (DCK#10) and control cells (NT1) identified that oxidative phosphorylation (OXPHOS) was increased in DCK#10 cells [10]; however, functional relevance of the increased OXPHOS remained unclear.

**Fig. 1:**
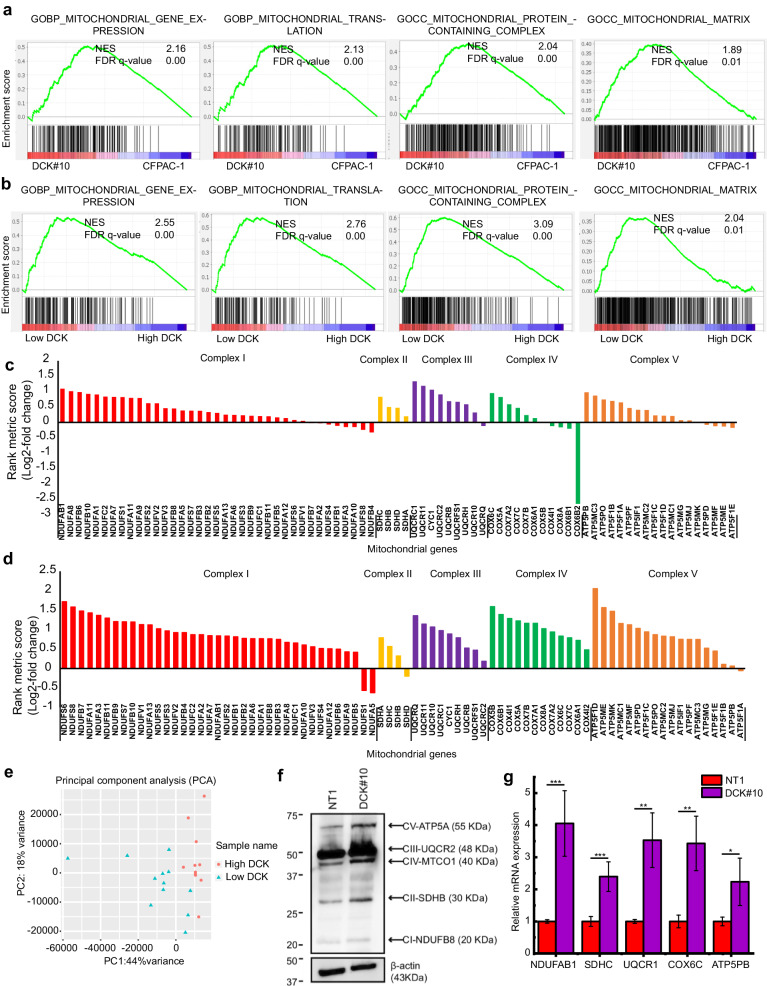
Deoxycytidine kinase (DCK) inactivation and lower DCK expression level are correlated with higher mitochondrial functions. **a**, **b** Gene Set Enrichment Analysis (GSEA) plots demonstrating a significant mitochondrial pathway enrichment (GOBP_MITOCHONDRIAL_GENE_EXPRESSION, GOBP_MITOCHONDRIAL_TRANSLATION, GOCC_MITOCHONDRIAL_PROTEIN_CONTAINING_COMPLEX, GOCC_MITOCHONDRIAL_MATRIX) in DCK#10 cells compared to parental CFPAC-1 cells (**a**) and in PDAC patients with low DCK expression compared to those with high DCK expression (**b**). **c**, **d** Bar graphs revealing the upregulation and downregulation of genes related to mitochondrial complexes I to V in DCK#10 (**c**) and low DCK-expressing PDAC patients (**d**). The Y axis represents the rank metric score in log2-fold ratio. **e** Principal component analysis of the genes belonging to HALLMARK_OXIDATIVE_PHOSPHORYLATION for 23 pancreatic cancer cases from The Cancer Genome Atlas. Using logarithmic values, gene expression data were analyzed. Sample name: High DCK, cases with high DCK expression; low DCK, cases with low DCK expression. Cases with low and high DCK expression are partially segregated by the expression profiles of the two groups. **f** Immunoblots showing the expression of five mitochondrial proteins (NDUFB8, SDHB, UQCRC2, MTCO1 and ATP5A) belonging to mitochondrial respiratory chain complexes (I, II, III, IV, V, respectively). β-actin; a loading control. **g** Reverse transcription-quantitative polymerase chain reaction (RT-qPCR) demonstrating the expression levels of the representative genes for mitochondrial complexes I to V in DCK#10 and control NT1 cells. Data are expressed as mean ± standard deviation from three independent triplicate experiments. *P < 0.05; **P < 0.01; ***P < 0.001.

GSEA was conducted to investigate the relationship between DCK inactivation and mitochondria function in DCK#10 cells. We found that GOBP_mitochondrial_gene_expression, GOBP_ mitochondrial_translation, GOCC_mitochondrial_protein_containing_complex, and GOCC_ mitochondrial_matrix gene sets were considerably upregulated in DCK#10 cells compared with NT1 cells (Fig. 1a). In parallel, TCGA PDAC datasets (paad_ tcga_pan_can_atlas_2018) were analyzed to examine the clinical relevance of a lower DCK expression and mitochondrial function in PDAC patients. We divided the patients into two groups based on the DCK mRNA expression levels (high DCK, *n* = 11 [Z-score > 1.25]; low DCK, *n* = 12 [Z-score < −1.25]). The same gene sets were upregulated significantly in the patient group with a lower DCK expression (Fig. 1b). We also noted that most genes encoding each mitochondrial complex I through V were upregulated in DCK#10 cells compared to NT1 cells (Fig. 1c). Consistently, PDAC patients with a lower DCK expression demonstrated increased gene expression of all mitochondrial complexes (Fig. 1d). Principle component analysis revealed that genes belonging to hallmark OXPHOS and GOBP mitochondrial expression separated the patient clusters with low and high DCK expression (Fig. 1e and Supplementary Fig. S1a). Additionally, the GOBP_mitochondrial_gene_expression gene set was substantially elevated in patients with a low DCK (Supplementary Fig. S1b). To examine mitochondrial protein expression, we conducted Western blotting (WB) using an antibody cocktail for mitochondrial OXPHOS proteins. The results revealed upregulation of NDUFB8, SDHB, UQCRC2, MTCO1 and ATP5A proteins in DCK#10 cells (Fig. 1f). For further confirmation, the mRNA expression levels were examined using real-time quantitative RT-PCR (RT-qPCR) in cultured DCK#10 and NT1 cells and in xenograft tumors established from DCK#10 and NT1 cells. Among the mitochondrial complex genes, *NDUFAB, SDHC*, *UQCR1*, *COX6C*, and *ATP5PB* were all upregulated in DCK#10 cells in vitro culture condition (Fig. 1g). Likewise, all genes except *ATP5PB* were significantly upregulated in the DCK#10 xenografted tumors (Supplementary Fig. S1c). These findings suggest that DCK inactivation is associated with higher mitochondrial complex gene expression.

### DCK-deficient PDAC cells have higher OXPHOS

**Fig. 2:**
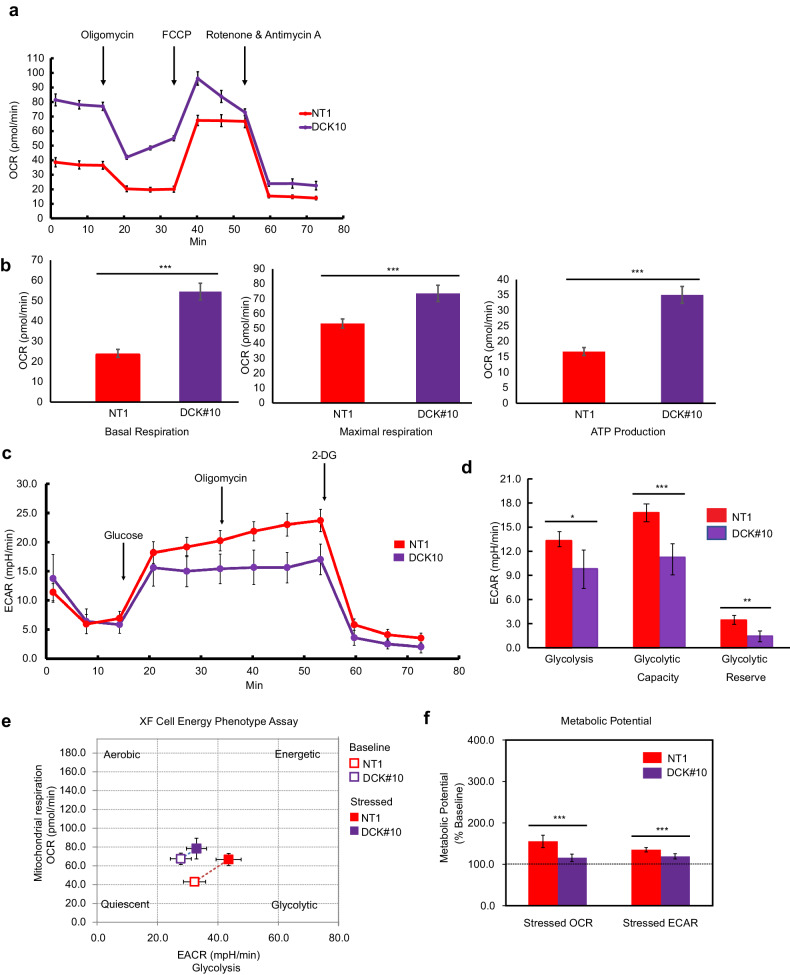
DCK-inactivated PDAC cells have a higher oxidative phosphorylation. **a** Mito stress test profiles demonstrating an increase in oxygen consumption rate (OCR) in DCK#10 under basal condition, followed by the injection of oligomycin (1.5 μmol/L), FCCP (1 μmol/L), and rotenone + antimycin A (0.5 μmol/L). **b** Bar graphs revealing increased basal respiration, maximal respiration, and ATP production rate in DCK#10 cells in (**a**). **c** Glycolysis stress test profiles demonstrating a decrease in extracellular acidification rate (ECAR) in DCK#10 under basal condition, followed by the injection of glucose (10 mmol/L), oligomycin (1 μmol/L), and 2-deoxyglucose (50 mmol/L). **d** Bar graph demonstrating reduced glycolysis, glycolytic capacity, and glycolytic reserve in DCK#10 cells in (**d**). **e** Energy map revealing reduced DCK#10 cell energetic efficiency under both baseline and stressed conditions. **f** Reduced metabolic potential of DCK#10 cells compared to control NT1 cells under energetically stressed conditions. NT1, *n* = 10; DCK#10, *n* = 8 for (**e**) and (**f**). Data are expressed as mean ± standard deviation from three independent triplicate experiments. *P < 0.05; **P < 0.01; ***P < 0.001.

We conducted extracellular flux analysis using Seahorse XFe system to evaluate metabolic function in living cells. Concurring with the upregulation of mitochondrial complex genes, we found that the basal OCR was remarkably higher in DCK#10 cells compared to the control NT1 cells (Fig. 2a). DCK#10 cells also revealed a higher maximal respiration capacity and mitochondrial ATP production (Fig. 2b), which indicates that DCK inactivation improves mitochondrial metabolic activity. Conversely, reintroducing wild-type DCK in DCK#10 cells (HA-DCK) significantly lowered OCR and ATP production compared to the control (EV) cells, while the kinase activity-deficient mutant (HA-DCK-KD) also slightly induced the similar effect. (Supplementary Fig. S2a, b). This suggests that DCK knockout enhances mitochondrial OXPHOS in both its kinase activity-dependent and -independent manners. On the other hand, we noted reduced glycolysis, glycolytic capacity, and glycolytic reserve in DCK#10 cells as shown by the lower ECAR (Fig. 2c, d). These data suggest that the ATP production in DCK-deficient cells mostly depends on OXPHOS. Next, the influence of DCK inactivation on the cellular glycolytic and mitochondrial metabolic potential was simultaneously assessed (i.e., metabolic flexibility) under energetically stressed conditions. While DCK#10 cells were more aerobic as expected, those were less metabolically flexible compared to NT1 cells (Fig. 2e and Supplementary Fig. S2c, d). In DCK#10 cells, the metabolic potential was reduced as demonstrated by the stressed OCR and ECAR (Fig. 2f). In support of these findings, we noted less mitochondrial spare capacity in DCK#10 cells compared to NT1 cells, which refers to the decreased mitochondrial respiration capacity in handling additional energy demand or stress beyond their baseline requirements (Supplementary Fig. S2e). Hence, DCK inactivation leads to the notable energetic shift from glycolysis to OXPHOS.

### DCK inactivation induces abnormal mitochondrial morphology in PDAC cell line

**Fig. 3:**
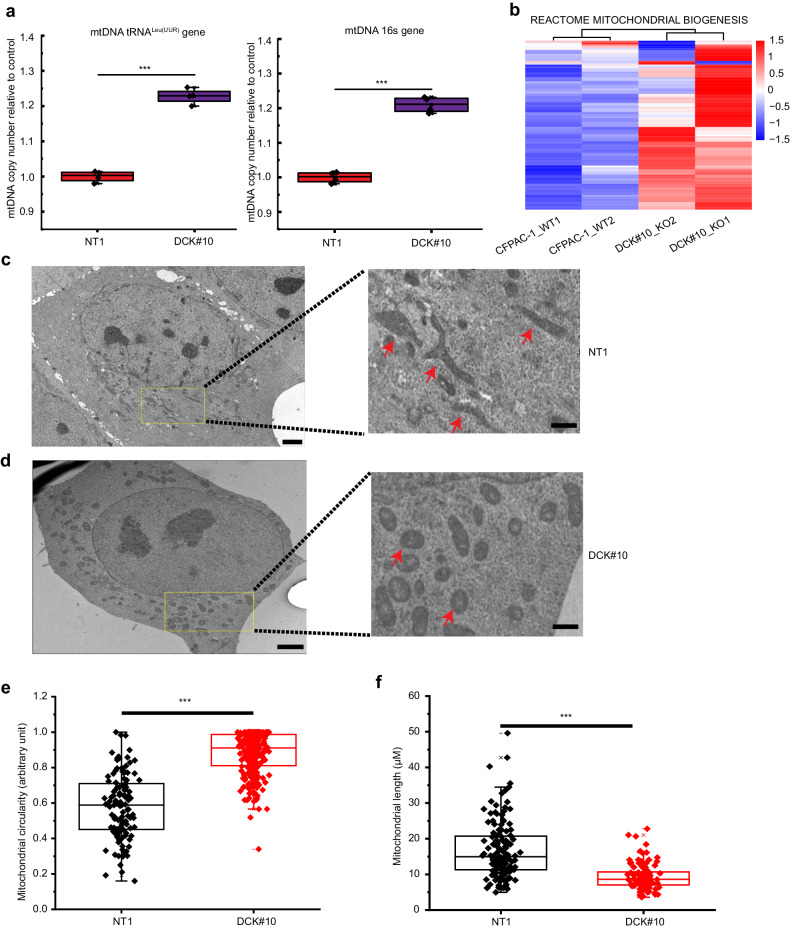
DCK inactivation alters mitochondrial morphology in PDAC cell. **a** Quantitative PCR analysis demonstrating mtDNA copy number of mitochondrial-encoded tRNA^Leu(UUR)^ and mtDNA 16S rRNA gene over nuclear-encoded B2M. **b** Heatmap demonstrating a clustered gene expression based on the genes belonging to the REACTOME_MITOCHONDRIAL_BIOGNESIS gene set. The corresponding heatmap of the barcode representation is shown to the right of the heatmap. Red and blue correspond to high and low expression levels. Each group comprised of two biological replicates. Representative images of transmission electron microscopy for control NT1 (**c**) and DCK#10 cells (**d**). Left panels: low magnification, scale bar, 25 μm. Right panels: high magnification of the yellow rectangular areas in the left panels, scale bar, 5 μm. Arrows indicate examples of mitochondria. Box plots demonstrating the average mitochondrial circularity (**e**) and length (largest side of mitochondria) (**f**) in DCK#10 and NT1 cells. The box extends from the 25th–75th percentile; the lines within the middle of the box constitute the median of the values. The whiskers represent the 10th–90th percentiles. Outliers are indicated as cross (×) and minus ($-$) (**e**) NT1, *n* = 188; DCK#10, *n* = 277 and (**f**) NT1, *n* = 129; DCK#10, *n* = 106. Data are from three independent triplicate experiments. *P < 0.05; **P < 0.01; ***P < 0.001.

We measured the copy number of the mitochondrial gene tRNA^Leu(UUR)^ and mitochondrial DNA (mtDNA) 16S rRNA normalized to the nuclear gene β-2-microglobulin to further characterize the impact of DCK inactivation on mitochondria (Fig. 3a). The mtDNA copy number was upregulated significantly in DCK#10 cells relative to the NT1 cells. Consistently, the genes responsible for mitochondrial biogenesis (Reactome Mitochondrial Biogenesis gene set) were upregulated in DCK-deficient cells (Fig. 3b). Additionally, the mRNA expression of mitochondrial fusion genes including MFN1, MFN2, and OPA1, along with fission gene DNM1L, exhibited increased levels in DCK#10 cells and in xenografts derived from DCK#10 cells. These findings suggest enhanced mitochondrial dynamics upon DCK inactivation (Supplementary Fig. S3a, b). Mitochondrial morphology using transmission electron microscopy was examined next. Most mitochondria in the DCK#10 cells were rounded to oval with a less dense matrix compared to the NT1 cells, where mitochondria have a tubular morphology (Fig. 3c, d). We measured the circularity and the length of the longest side of the mitochondria to further verify the morphological difference. We noted that mitochondria became more circular on DCK inactivation (Fig. 3e and Supplementary Fig. S3c, d), whereas mitochondria became elongated in NT1 cells (Fig. 3f and Supplementary Fig. S3e, f). A rounded mitochondrial morphology with a higher basal OCR and ATP production was previously reported in aggressive PDAC cell lines [20]. Hence, the morphological changes in DCK-deficient cells may also be associated with a higher OXPHOS although molecular mechanisms underlying these findings need to be addressed in future work.

### *SOD2* provides stability for DCK-deficient PDAC cells

**Fig. 4:**
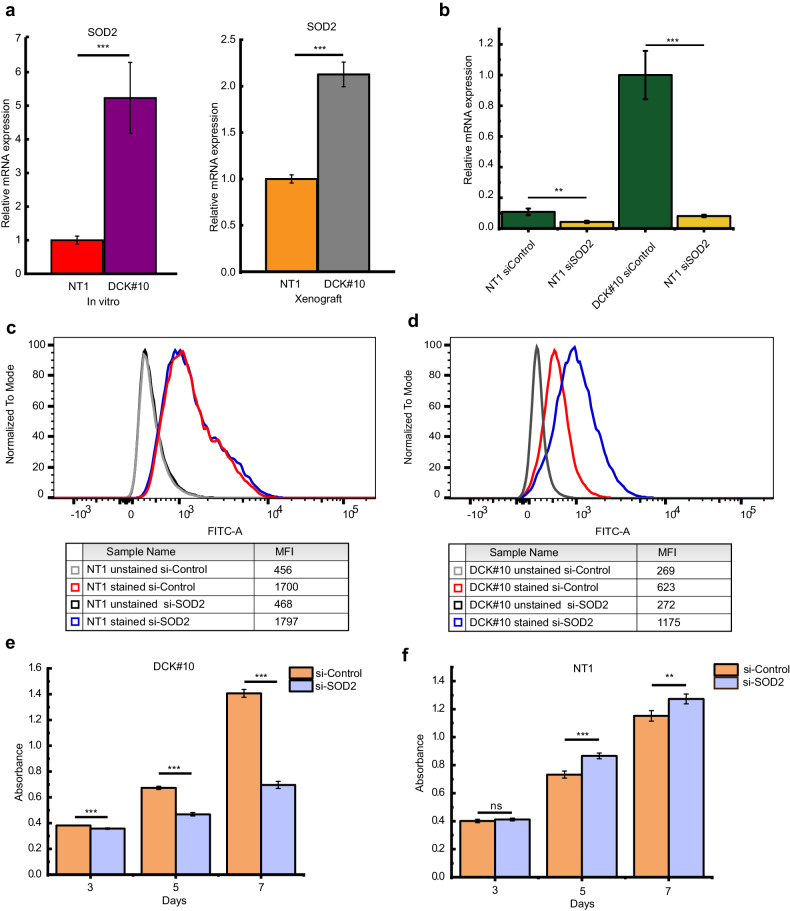
SOD2 provides DCK-inactivated PDAC cell stability. **a** RT-qPCR showing cellular ROS scavenger gene expression, *SOD2* gene, in NT1 and DCK#10 cells in vitro (left) and in xenograft tumor from NT1 and DCK#10 cells (right). **b** RT-qPCR results showing *SOD2* gene expression following a 72-h transfection of siControl and siSOD2 RNAi into NT1 and DCK#10 cells. **c**, **d** Flow cytometric plots showing the fluorescent intensity (FITC-A) of DCK#10 and NT1 cells following MitoSOX green (MSG) staining, an indicator of mitochondrial superoxide levels. MFI; mean fluorescence intensity. **e**, **f** Cell proliferation assay of DCK#10 (**e**) and NT1 (**f**) cells with siControl and siSOD2 RNAi transfection. Cell proliferation was measured at days 3, 5, and 7 after siRNA introduction using WST-8 cell counting kit. Data are expressed as mean ± standard deviation from three independent triplicate experiments. *P < 0.05; **P < 0.01; ***P < 0.001.

Generally, mitochondria with a high OXPHOS activity produce more ROS [30]. Surprisingly, however, FACS analysis demonstrated reduced levels of basal ROS in DCK#10 cells with higher OXPHOS activity than those of control NT1 cells (Supplementary Fig. S4a). Furthermore, DCK#10 cells demonstrated a smaller increase in intracellular ROS levels on H_2_O_2_ treatment compared to NT1 cells (Supplementary Fig. S4b, c), suggesting enhanced ROS scavenging capacity in DCK#10 cells. We examined the expression of *SOD1* (Cu/ZnSOD; superoxide dismutase 1) and *SOD2* (MnSOD; superoxide dismutase 2) genes, which respectively act as cytosolic and mitochondrial ROS scavenger in cells, to assess the underlying molecular mechanisms [31]. A higher *SOD1* and *SOD2* gene expression was observed in DCK#10 cells in both cultured DCK#10 cells and xenografted DCK#10 cells compared to those from control NT1 cells (Fig. 4a and Supplementary Fig. S4d). To test the possibility whether SOD2 provided survival advantage by counteracting increased ROS production in DCK#10, DCK#10 and NT1 cells were first transfected with siSOD2 and siControl (control siRNA) and knockdown of *SOD2* gene was confirmed (Fig. 4b). The knockdown of the mitochondrial ROS scavenging gene *SOD2* in NT1 cells did not show a noticeable increase in mitochondria-specific ROS levels (Fig. 4c). In contrast, a marked increase in these levels was observed in DCK#10 cells following *SOD2* knockdown (Fig. 4d). Then, the proliferation of DCK#10 and NT1 cells was examined following *SOD2* inactivation at days 3, 5, and 7. A significant reduction in the proliferation of DCK#10 cells with *SOD2* inhibition was observed (Fig. 4e). Conversely, the cytostatic effect of SOD2 inactivation was minimal in NT1 cells (Fig. 4f). These results suggest that *SOD2* is crucial for scavenging excess ROS to provide DCK#10 cell stability with a higher OXPHOS activity.

### DCK-deficient PDAC cells are sensitive to mitochondrial complex **I** and BCL2 inhibitors

**Fig. 5:**
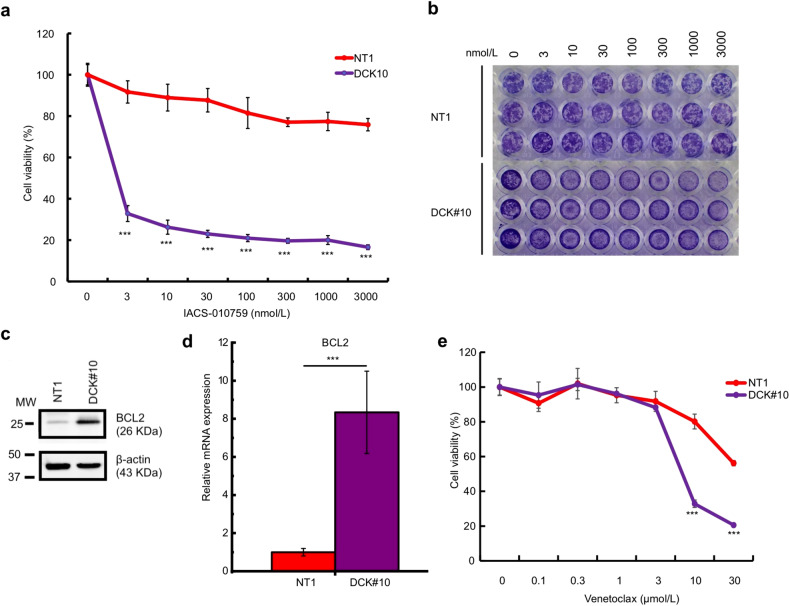
DCK-inactivated PDAC cells are sensitive to mitochondrial complex I and BCL2 inhibitors. **a** Dose–response curve of mitochondrial complex I inhibitor, IACS-010759 (0–3000 nmol/L) in DCK#10 and NT1 cells. Cells were treated with IACS-010759 for 72 h. Data are expressed as mean ± standard deviation from three independent triplicate experiments. **b** Representative crystal violet staining images demonstrating DCK#10 and NT1 cell viability after IACS-010759 treatment (0–3000 nmol/L). **c** Immunoblot showing elevated expression of BCL2 protein in DCK#10 cells compared to control NT1 cells. β-actin; a loading control. **d** RT-qPCR revealing anti-apoptotic gene expression, *BCL2*, in control NT1 and DCK#10 cells. **e** Dose-response curve of a BCL2 inhibitor, venetoclax (0–30 µmol/L) in DCK knockout (DCK#10) and control NT1 cells. Cells were treated with venetoclax for 72 h. Data are expressed as mean ± standard deviation from three independent triplicate experiments. *P < 0.05; **P < 0.01; ***P < 0.001.

The results of the GSEA and Seahorse experiments reveal the mitochondrial function upregulation in DCK#10 cells. Hence, whether the inactivation of mitochondrial complex I of the electron transport chain (ETC) affects the DCK#10 cell proliferation was tested. We selected an inhibitor of mitochondrial complex I, IACS-010759, because of its higher selectivity [32, 33]. We observed that DCK#10 cells were highly sensitive to IACS-010759 with an IC50 value of 1.75 ± 0.02 nmol/L compared to NT1 cells with an IC50 > 3 µmol/L (Fig. 5a). Since WST-8, a tetrazolium salt used for the proliferation assay, is reduced by cellular dehydrogenase and may be influenced by mitochondrial function, the effect of IACS-010759 using crystal violet staining was further validated, and the same conclusion with WST-8-based proliferation assay was obtained (Fig. 5b). Subsequently, based on the findings outlined in Figs. 2 and 4, we hypothesized that a higher OXPHOS leading to increased ROS production might indicate a potential correlation between DCK inactivation and mitochondrial membrane potential. Thus, DCK#10 cells maintained an equilibrium state of stability, reducing susceptibility to apoptotic cell death. To verify this idea, BCL2 protein levels and mRNA expression were analyzed, a crucial apoptotic inhibitor [34, 35], and found that BCL2 was significantly upregulated in DCK#10 cells compared to NT1 cells (Fig. 5c, d). We noted that DCK#10 cells were more sensitive to a BCL2 inhibitor, venetoclax, with an IC50 value of 7.85 ± 0.25 µmol/L compared to NT1 cells whose IC50 value was higher than 30 µmol/L (Fig. 5e). These findings show that inactivation of DCK in PDAC cells increases their dependency on the mitochondrial ETC and anti-apoptotic pathways in addition to gemcitabine resistance.

### Effect of mitochondrial complex **I** inhibitor on DCK-inactivated PDAC cells in vivo

**Fig. 6:**
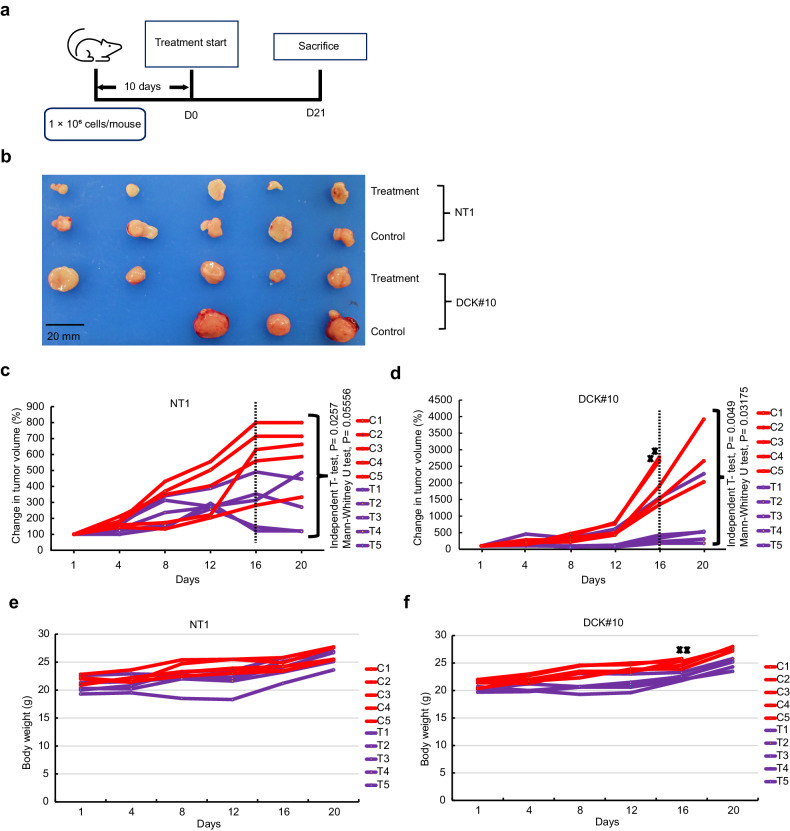
Effect of mitochondrial complex I inhibitor on DCK-inactivated PDAC in vivo. **a** Schematic representation of the experimental setup. In the right flank of 5-week-old BALB/cAJcl-nu/nu male nude mice, 1 million control NT1 and DCK#10 cells were transplanted. The treatment was initiated 10 days after transplantation (D0). The recipient mice were sacrificed 21 days after treatment (D21). **b** Representative images of the excised tumors at D21 derived from DCK#10 and NT1 cells in 0.5% methyl cellulose (control) and IACS-010759 treatment categories. Images of two tumors in control DCK#10 are not included since those tumors reached the upper limit of the volume prior to D21. **c**, **d** Plot demonstrating the relative tumor volume of NT1 (**c**) and DCK#10 (**d**) xenografts (tumor volume at D0 is set to 100%, respectively) treated with IACS-010759 (T) or 0.5% methyl cellulose (C; control) over day 21. Two mice from the DCK#10 control group were sacrificed at day 16 when the tumor volume reached the upper limit and are shown as cross (✖). The *P*-values were calculated at day 16, where *n* = 5 for both NT1 and DCK#10 xenograft tumors in both control and treatment categories. **e**, **f** Plot demonstrating the body weight of the 10 mice treated with 0.5% methyl cellulose (control) and IACS-010759 cohort of the NT1 (**e**) and DCK#10 xenograft (**f**) experiments during the 21st day of the experiment. C1-C5; control, T1-T5; treatment with 7.5 mg/kg IACS-010759.

**Fig. 7:**
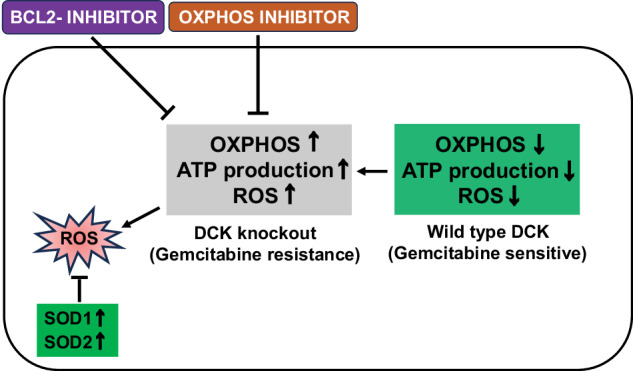
Schematic model displaying the metabolic reprogramming induced by DCK inactivation. One of the major factors contributing to gemcitabine resistance is DCK inactivation. Following DCK deletion, PDAC cells exhibited an enhanced reliance to OXPHOS, leading to an increased ATP production within the mitochondria. This metabolic shift renders the cells less glycolytic in nature. Moreover, DCK function loss leads to mitochondrial morphology alterations. Heightened dependence on OXPHOS results in elevated ROS production. To counteract the potential harmful effects of ROS, antioxidant levels of the enzymes SOD1 and SOD2 are increased in DCK-deficient PDAC cells, which stabilizes the cells and reduces the instability induced by the increased ROS.

To corroborate our in vitro findings, the effects of IACS-010759 in a xenograft model were evaluated. DCK#10 and NT1 cells were implanted in the right flank of BALB/c nude mice and the administration of IACS-010759 was initiated 10 days after the implantation (Fig. 6a). Tumor volumes were reduced during treatment with the mitochondrial complex I inhibitor in each group (Fig. 6b). We noted that DCK#10 cell-derived tumor had a higher proliferative capacity and resulted in larger tumor volumes compared to those derived from NT1 cells. Two of the DCK#10 cell-derived tumors in the untreated group arrived at the predetermined tumor volume limit, leading to the sacrifice of the mice (Supplementary Fig. S4e, f). Hence, tumor volumes on day 16 of IACS-010759 treatment were compared, where *n* = 5 for each group. In response to IACS-010759 treatment, DCK#10 cell-derived tumors revealed a significant reduction in volume compared to NT1 cell-derived tumors (*p* = 0.03175) as determined by non-parametric Mann–Whitney–Wilcoxon test (Fig. 6c, d). Throughout the experimental duration, no significant adverse effects were observed in mice. This was assessed through weight loss, longevity, behavior, and feeding habit monitoring, and no observable changes or concerns were detected (Fig. 6e, f). These results provide a strong evidence supporting the idea that gemcitabine-resistant PDAC cells induced by DCK inactivation can be targeted through mitochondrial complex I inhibition. In conclusion, the findings indicate that cells with DCK inactivation depend significantly on active mitochondrial function. Thus, targeting mitochondrial function appears as a potential strategy to overcome gemcitabine resistance in PDAC secondary to DCK inactivation (Fig. 7).

## Discussion

Gemcitabine monotherapy remains a standard treatment for PDAC [6, 36]. However, gemcitabine-based PDAC treatment is frequently hampered by chemoresistance development to this drug [37, 38]. Gemcitabine resistance is attributed to multiple mechanisms [38]. Among them, inactivating *DCK* mutations have been identified in various cancer cell lines and acute myeloid leukemia patients that are resistant to pyrimidine nucleoside analogs [39–42]. Additionally, we and others have reported that loss or reduced DCK expression is strongly correlated with gemcitabine resistance of PDAC cells and patient prognosis [9, 10]. Hence, the identification of cellular vulnerabilities secondary to DCK inactivation may lead to novel therapeutic approaches for gemcitabine-resistant PDAC.

In this study, *DCK* inactivation was found to be associated with increased mitochondria gene expression (Fig. 1) and is involved in increased OXPHOS activity and metabolic reprogramming (Fig. 2). Moreover, mitochondrial biogenesis and morphological changes are also associated with DCK inactivation (Fig. 3). Similar circular mitochondrial morphology with higher mitochondrial mass and OXPHOS were previously reported in a mouse pancreatic cancer model caused by Ras and Trp53 mutations [20]. The underlying molecular mechanisms remain unclear, although gemcitabine-resistant PDAC cells have been reported to have high OXPHOS activity [21, 43]. Here, our results strongly indicate that DCK inactivation contributes directly or indirectly to the increased OXPHOS activity. When DCK was ectopically expressed in gemcitabine-sensitive CFPAC-1 and gemcitabine-resistant HPAF-II cell lines, we observed no consistent reduction in the expression of mitochondrial genes that were elevated due to DCK knockout (Supplementary Fig. 5). These findings suggest that DCK might not act as a universal regulator of mitochondrial gene expression. Instead, its effects seem to vary depending on cellular contexts. However, future investigation is required to fully elucidate these issues. Notably, it was previously reported that MYC stimulates mitochondrial genes and mitochondrial biogenesis [44–46]. Consistent with these reports, the *MYC* gene signature is enriched in DCK-deficient PDAC cells as we previously elucidated [10].

Currently, IACS-010759, a mitochondrial complex I inhibitor, is in clinical trials in a variety of cancer types owing to its higher selectivity and better patient tolerability. Despite these advantages, lactic acidosis and neurotoxicity are the most common side effects in clinical trials [32, 33], underscoring the need for careful monitoring and management in its clinical application. Using patient-derived xenograft models and cell lines, higher MYC amplification was demonstrated to be a strong indicator of IACS-010759-sensitive BCs [47]. Furthermore, IACS-010759 efficiently eliminated tumors with defects in glycolysis [48]. We noted marked selectivity and sensitivity in DCK-KO PDAC cells compared to control cells (Fig. 5). Oral IACS-010759 administration significantly inhibited DCK-KO tumor growth in a xenograft model (Fig. 6). These findings concur with our finding that DCK-KO PDAC cells demonstrate a higher MYC activity [10] and low glycolysis **(**Fig. 2**)**, further supporting the rationale to target the OXPHOS pathway to eliminate gemcitabine-resistant PDAC cells induced by DCK inactivation.

Mitochondrial ROS production can increase with OXPHOS activity changes [49]. Excessive ROS levels potentially induce cellar damages [50, 51]. To maintain cellular redox balance, cells have evolved mechanisms to prevent oxidative damage, which include non-enzymatic and enzymatic antioxidants such as glutathione peroxidases, catalase, thioredoxin, and superoxide dismutases (SODs) [52–55]. Additionally, an anti-apoptotic gene, *BCL2*, also plays an important role in maintaining mitochondrial membrane potential and integrity to protect cells from excessive ROS levels [34, 35]. We found increased *SOD1*, *SOD2*, and *BCL2* expression and decreased intracellular ROS levels in DCK-deficient PDAC cells (Fig. 4). Consequently, siRNA introduction against *SOD2* and a BCL2 inhibitor, venetoclax, suppressed cell proliferation in DCK-deficient PDAC cells compared to control cells (Fig. 5). Venetoclax has also been reported to have an inhibitory effect on OXPHOS [56].

To fully exploit the therapeutic potential of complex I inhibitors in gemcitabine-resistant PDAC cells induced by DCK inactivation, additional work is warranted. Overtime, fine metabolomic analysis may reveal how DCK inactivation results to cellular metabolism reprograming. Nevertheless, our study demonstrated a significant relationship between DCK inactivation and higher OXPHOS activity in PDAC cells. We also identified *BCL2* and *SOD2* as essential, at least a part, in maintaining redox balance in DCK-inactivated PDAC cells with elevated OXPHOS activity. Targeting mitochondrial complex I may have additional clinical benefits due to the close relationship between increased OXPHPOS activity and metastatic potential [57, 58]. Although the detailed mechanisms by which DCK inactivation mediates metabolic reprogramming remain to be elucidated, targeting gemcitabine-resistant DCK-inactivated cells with OXPHOS inhibitors may become a spring board for new therapeutic avenues to overcome gemcitabine resistance in PDAC.

### Supplementary information

Supplementary file

Reproducibility checklist

## Data Availability

RNA-seq data obtained in this study have been deposited under the following accession numbers: experiment DRX370437-DRX370440 in the DDBJ (DNA Data Bank of Japan).
