# Correction: Deoxycytidine kinase inactivation enhances gemcitabine resistance and sensitizes mitochondrial metabolism interference in pancreatic cancer

**DOI:** 10.1038/s41419-024-06628-3

**Published:** 2024-05-31

**Authors:** Suman Dash, Takeshi Ueda, Akiyoshi Komuro, Masahiko Honda, Ryoichi Sugisawa, Hitoshi Okada

**Affiliations:** 1 Department of Biochemistry, Kindai University Faculty of Medicine, Osakasayama, Osaka 589-8511 Japan; 2 Graduate School of Medical Sciences, Kindai University Faculty of Medicine, Osakasayama, Osaka 589-8511 Japan; 3 Anti-aging Center, Kindai University, Higashi-Osaka, Osaka 577-8502 Japan

**Keywords:** Cancer metabolism, Cancer therapeutic resistance

Correction to: *Cell Death & Disease* 10.1038/s41419-024-06531-x, published online 12 February 2024

We wish to address errors in the original data, specifically the full crop western blot images for Fig. 1f (right panel), Fig. 5c, Fig. S5a (right panel) and S5c (right panel), provided in the supplementary materials of our paper. The deformities in the bands and boxes were inadvertently introduced during the file compiling process. Enclosed are the corrected versions of these images, ensuring the integrity of the data presented. This correction does not alter any results or conclusions of our paper. We apologize for any confusion and appreciate the community’s understanding.

Incorrect Original Data:
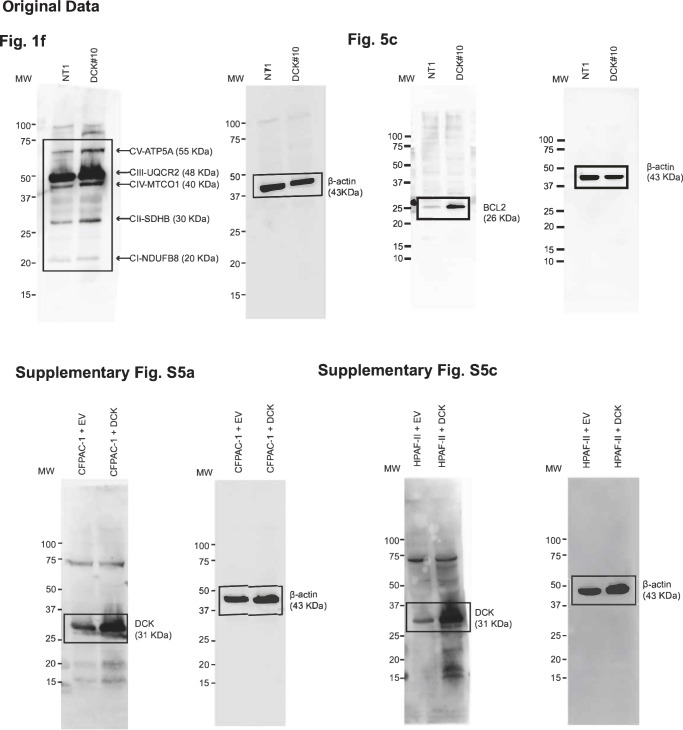


Updated Original Data:
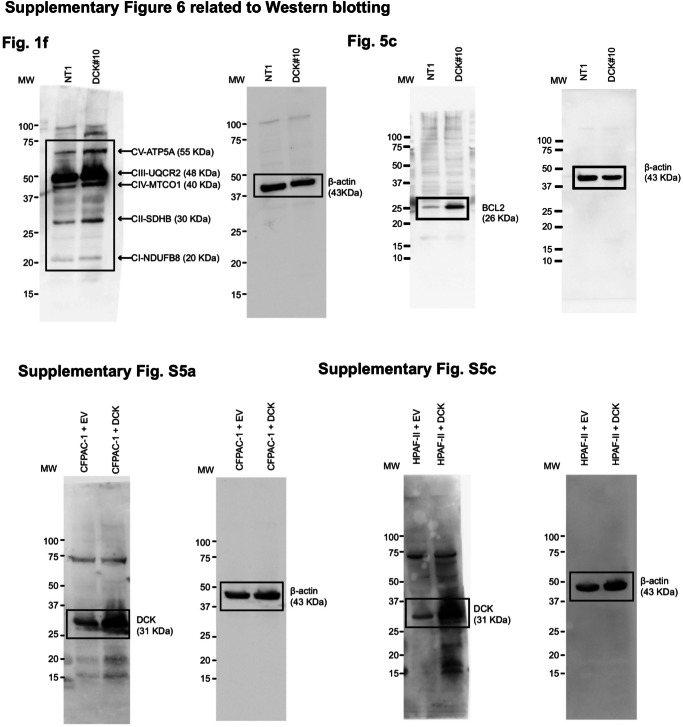


The original article has been corrected.

### Supplementary information

Supplementary information

