# Supplementary material for: Correction: Deoxycytidine kinase inactivation enhances gemcitabine resistance and sensitizes mitochondrial metabolism interference in pancreatic cancer
Source: Cell Death Dis. 2024 May 31;15(5):384. doi: 10.1038/s41419-024-06628-3 (PMC11143352; doi:10.1038/s41419-024-06628-3)
Supplement: Supplementary file 1 — Supplementary information [file 41419_2024_6628_MOESM1_ESM.pdf]

Supplementary Figure S1

Dash et al. supplementary Figure S1

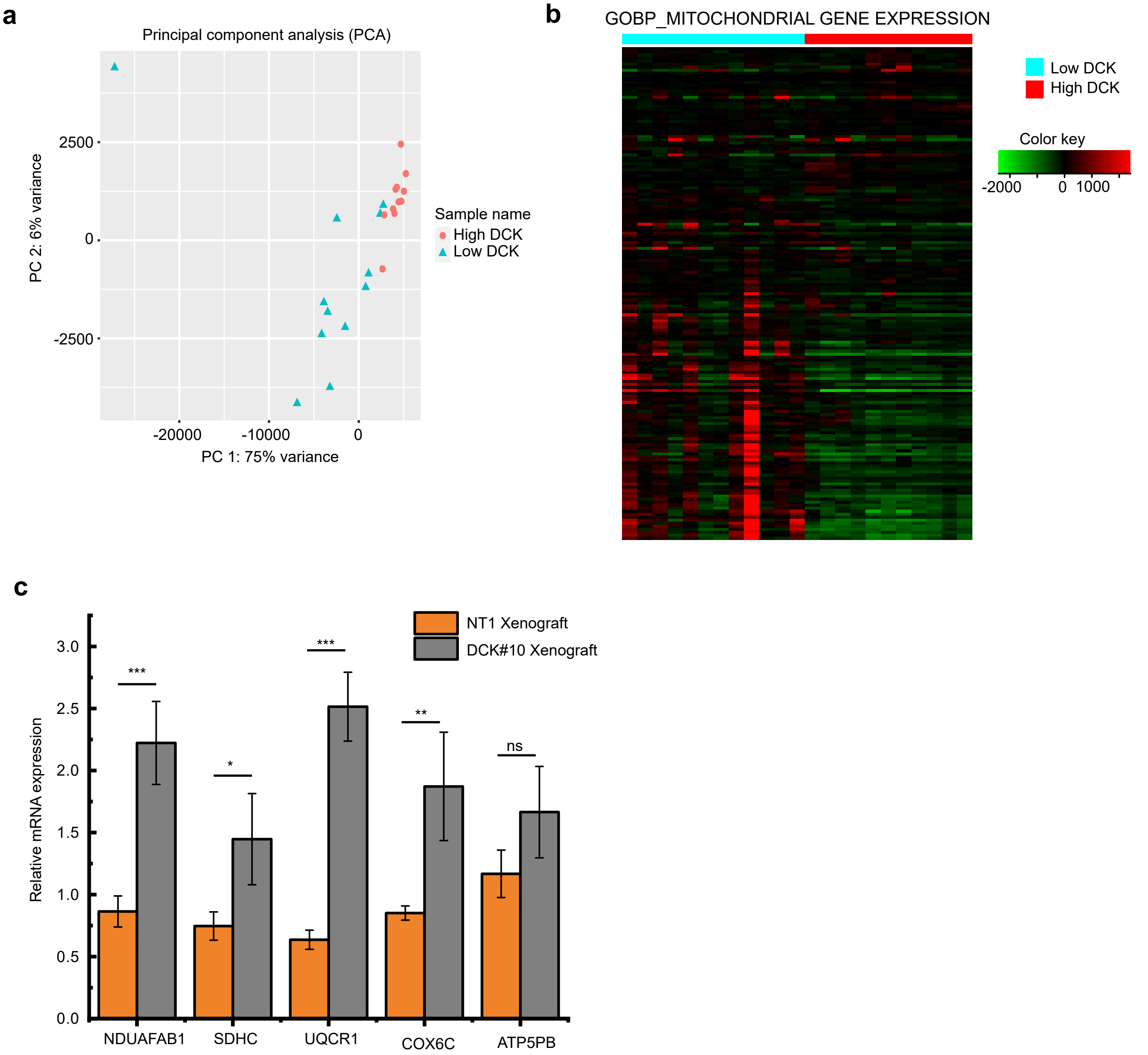

Supplementary Figure S1.
Supplementary results corresponding to Figure 1
a) Principal component analysis of the genes belonging to GOBP_MITOCHONDRIAL_GENE_EXPRESSION for the 23 pancreatic cancer cases. Gene expressions were analyzed as logarithmic values. Sample name: high DCK, cases with high DCK expression; low DCK, cases with low DCK expression. Cases with low and high DCK expressions are partially segregated by the gene expression profiles of the two groups. **b)** Heatmap revealing genes expressions belonging to GOBP_MITOCHONDRIAL_GENE_EXPRESSION gene set. **c)** RT-qPCR showing the expression of the representative genes for the mitochondrial complexes I to V in
DCK#10 and control NT1-derived xenograft. Data are expressed as mean ± standard deviation from three independent triplicate experiments. *P < 0.05; **P < 0.01; ***P < 0.001; ns, not significant. RT-qPCR, real-time quantitative reverse transcription polymerase chain reaction.

Supplementary Figure S2

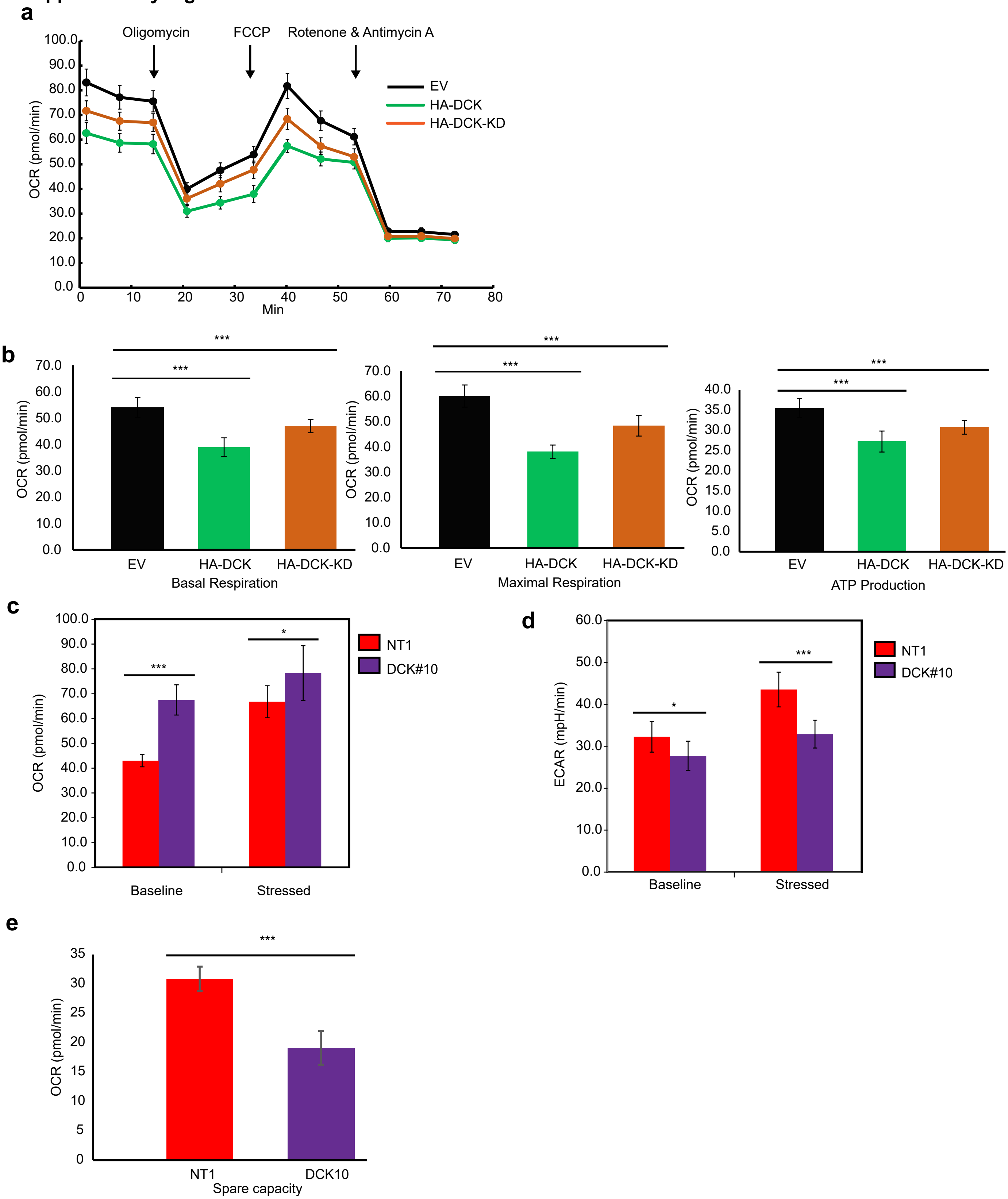

Supplementary Figure S2

Supplementary results corresponding to Figure 2

a and b) Mito stress test profiles demonstrating reduced oxygen consumption rate (OCR) in HA-DCK and HA-DCK-KD cells under basal condition, followed by the injection of oligomycin (1.5 µmol/L), FCCP (1 µmol/L), and rotenone + antimycin A (0.5 µmol/L). b) Bar graphs revealing basal respiration, maximal respiration, and ATP production rate in EV, HA-DCK and HA-DCK-KD cells (n=10). **c and d)** Cell energy phenotype assay demonstrating the baseline and stressed OCR (c) and ECAR (d) in DCK#10 and NT1 cells. (c) and (d) for NT1, n = 10; DCK#10, n = 8. **e)** Mito stress assay revealing the reduced spare capacity of the DCK#10 cell compared to the control NT1 cells. Data are expressed as mean ± standard deviation from three independent triplicate experiments. *P < 0.05; **P < 0.01; ***P < 0.001. EV; empty vector, HA–DCK; HA-tagged wild-type DCK, HA-DCK–KD; HA-tagged kinase-dead mutant of DCK.

Supplementary Figure S3

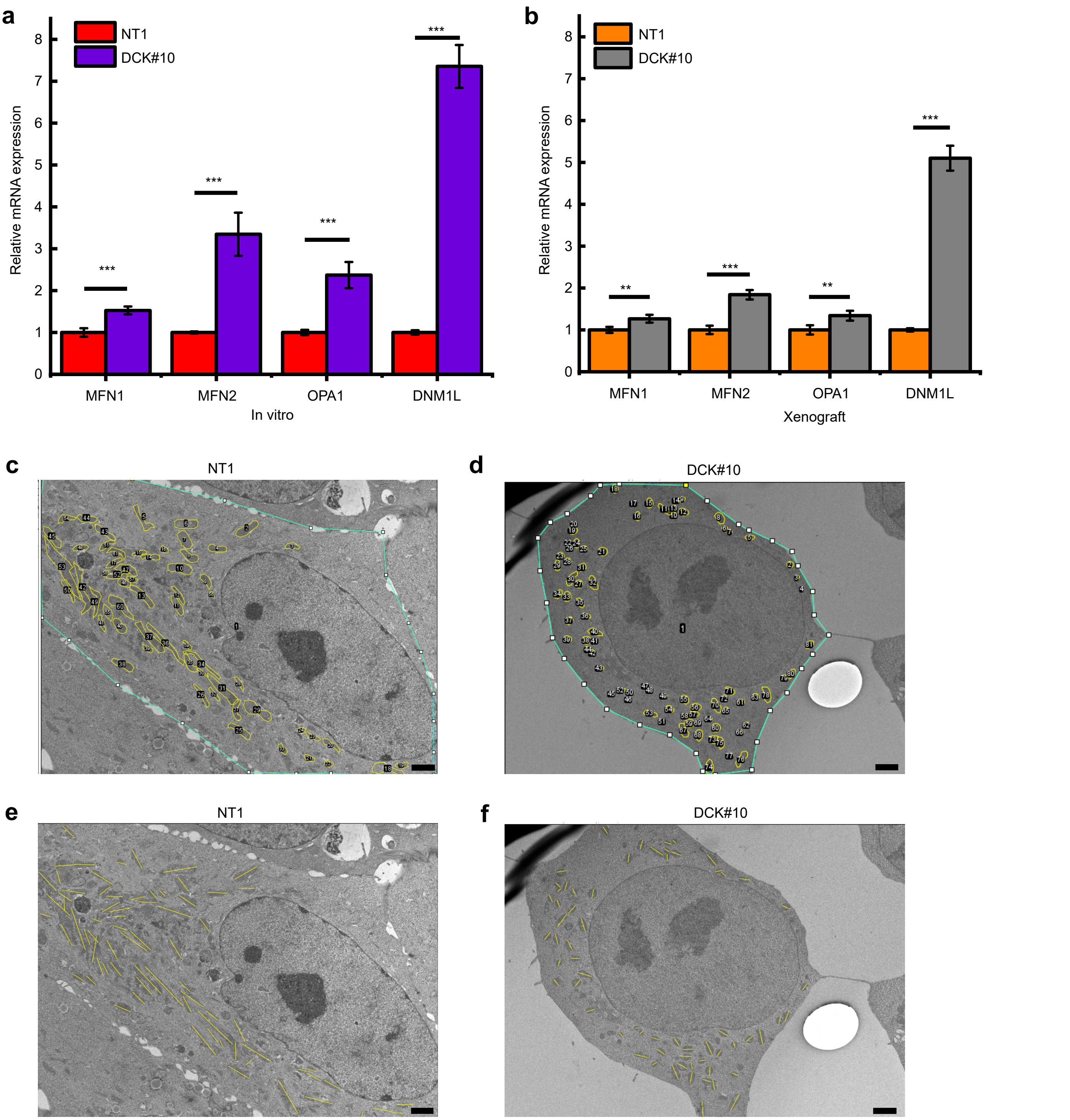

Supplementary Figure S3
Supplementary results corresponding to Figures 3
a and b) RT-qPCR showing the expression of the representative genes for mitochondrial fusion (MFN1, MFN2 and OPA1) and fission (DNM1L) pathways in NT1 and DCK#10 cells in vitro (left) and in xenograft tumor derived from NT1 and DCK#10 cells (right). **c–f)** Representative quantification of mitochondrial circularity (c and d) and length (e and f) from transmission electron microscopy images at 1000× magnification in control NT1 cell (c and e) and DCK#10 cell (d and f). Using ImageJ software, circularity and length of the mitochondria were measured. One mitochondrion is represented by each yellow closed shape in c and d, and yellow line in e and f, circularity and length, respectively. Scale bar, 20 μm.

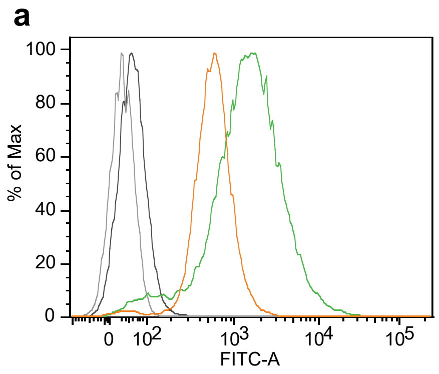

Sample Name	MFI
NT1 unstained	59.4
DCK#10 unstained	30.9
NT1 stained	2181
DCK#10 stained	646

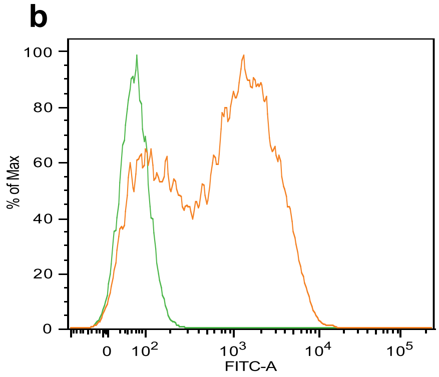

Sample Name	MFI
NT1_H2O2_Unstained	69.5
NT1_H2O2_stained	1335

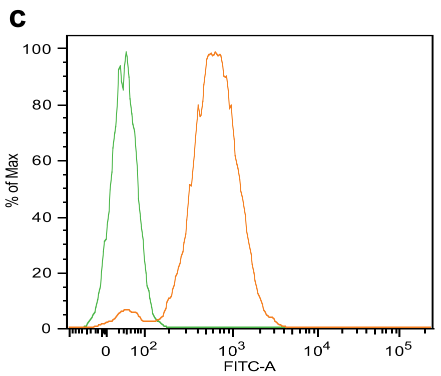

Sample Name	MFI
DCK#10_H2O2_Unstained	42.8
DCK#10_H2O2_stained	731

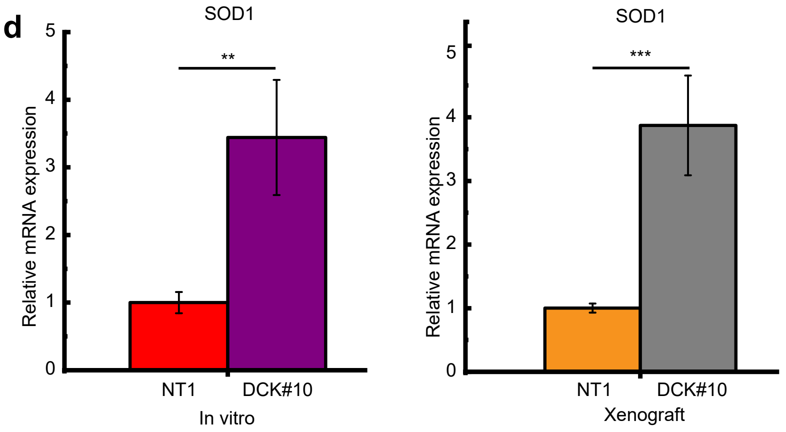

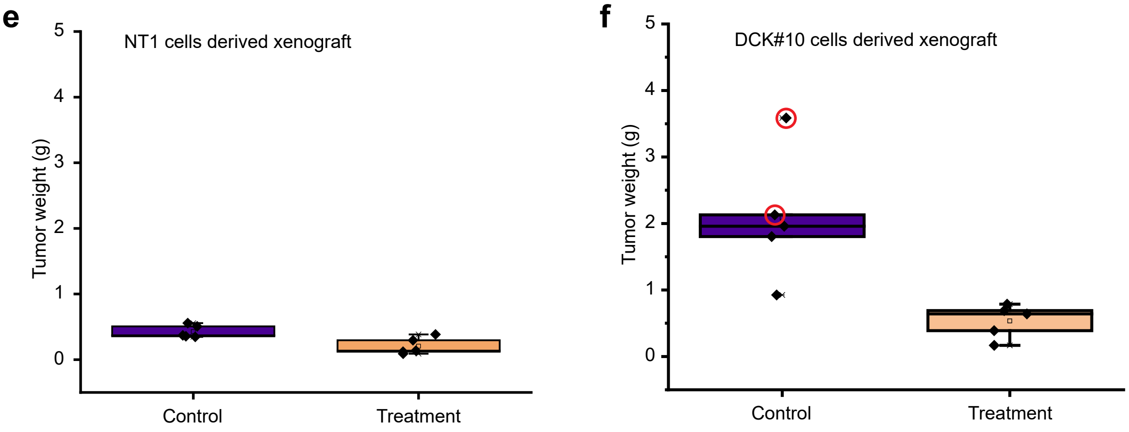

Supplementary Figure S4
Supplementary results corresponding to Figure 4 and Figure 6
a) Flow cytometric plot showing the fluorescent intensity (FITC-A) of DCK#10 and NT1 cells following H2DCFDA staining, an indicator of intracellular ROS levels. MFI; mean fluorescence intensity. **b and c)** FACS data showing the fluorescent intensity (FITC-A) of NT1 (b) and DCK#10 (c) cells after 1-h treatment with 250 µmol/L H2O2 followed by 1-h staining with 2 µmol/L H2DCFDA in the dark at 37°C. **d)** RT-qPCR showing cellular ROS scavenger gene expression, SOD1 gene, in NT1 and DCK#10 cells in vitro (left) and in xenograft tumor from NT1 and DCK#10 cells (right). **e and f)** Boxplots demonstrating the tumor weight of NT1 and DCK#10 xenografts treated with IACS-010759 and 0.5% methyl cellulose (control) at day 21. Two mice from DCK#10 group were sacrificed 16 days after treatment initiation and are indicated as red circles.

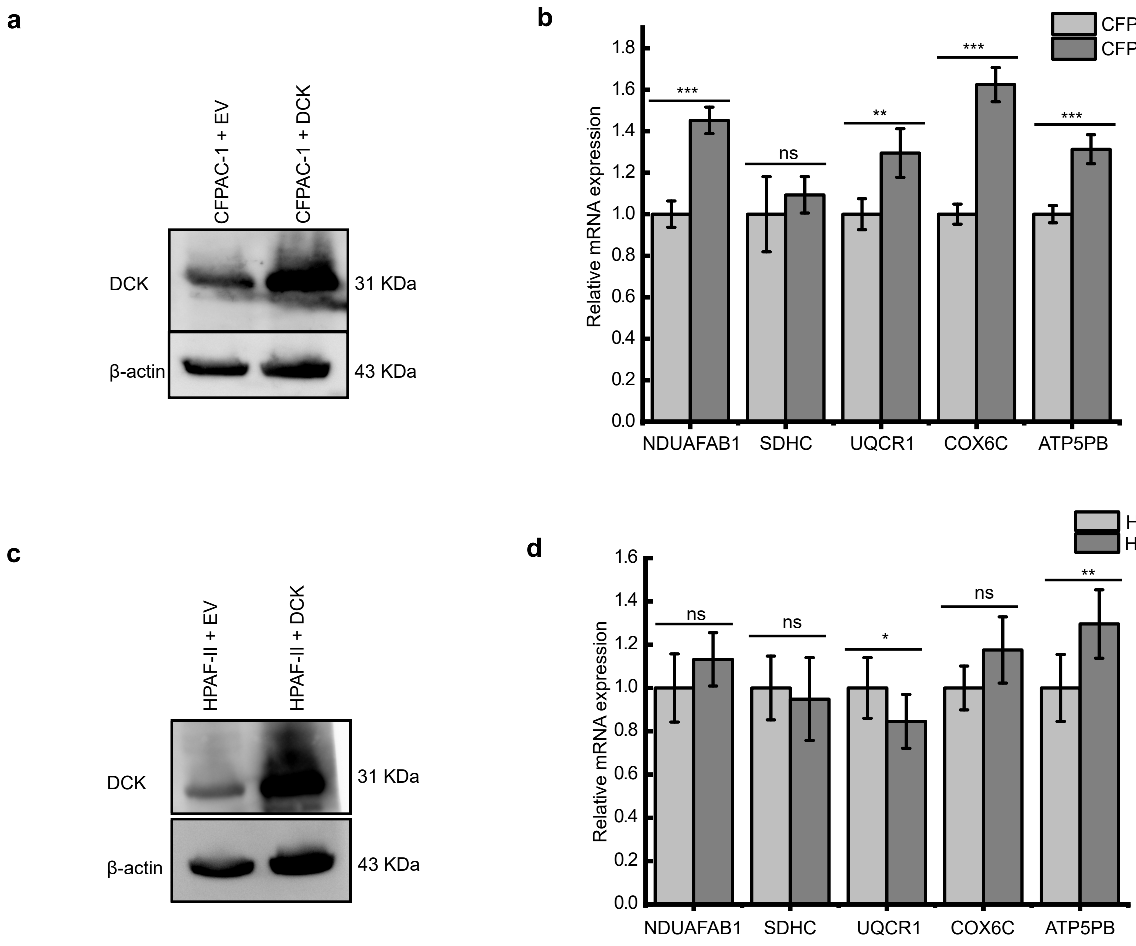

Supplementary Figure 5

DCK ectopic expression in CFPAC-1 and HPAF-II cell lines

a-d) Immunoblot showing protein expression of DCK in (a) CFPAC-1 and (c) HPAF-II cell lines. RT-qPCR demonstrating the expression levels of the representative genes for mitochondrial complexes I to V in CFPAC-1 + EV, CFPAC-1 + DCK (b); HPAF-II + EV and HPAF-II+ DCK cells (d). Data are expressed as mean ± standard deviation from three independent triplicate experiments. *P < 0.05; **P < 0.01; ***P < 0.001. CFPAC-1 + EV; CFPAC-1 cells with empty vector, CFPAC-1 + DCK; CFPAC-1 cells with wild-type DCK, HPAF-II + EV; HPAF-II cells with empty vector, HPAF-II + DCK; HPAF-II cells with wild-type DCK.

Original Data

Fig. 1f

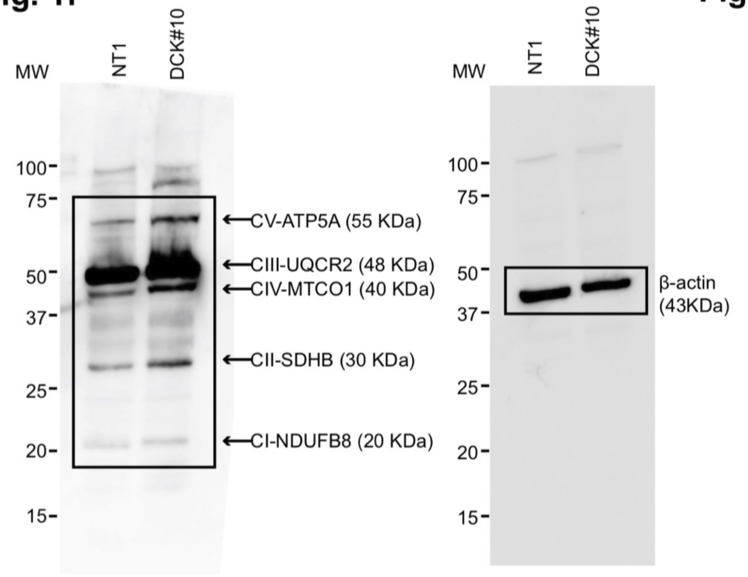

Fig. 5c

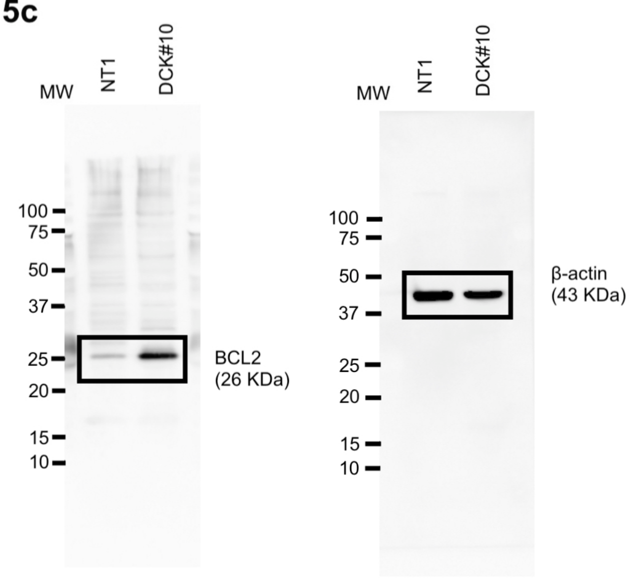

Supplementary Fig. S5a

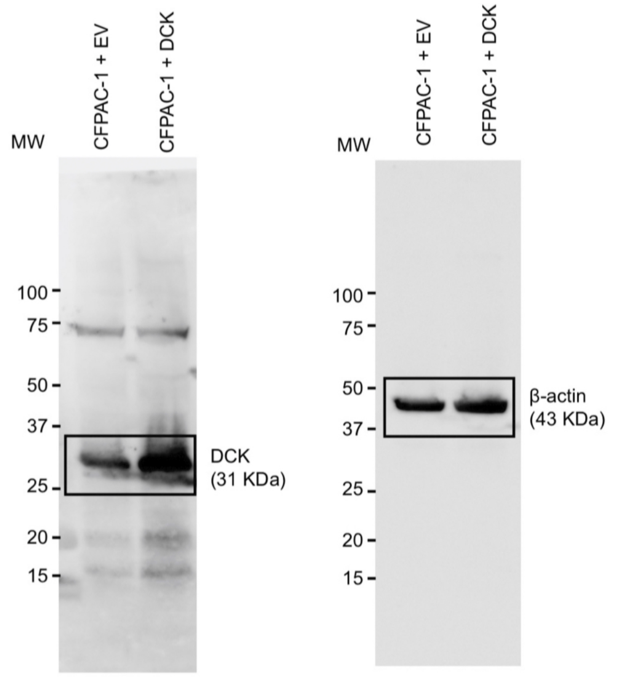

Supplementary Fig. S5c

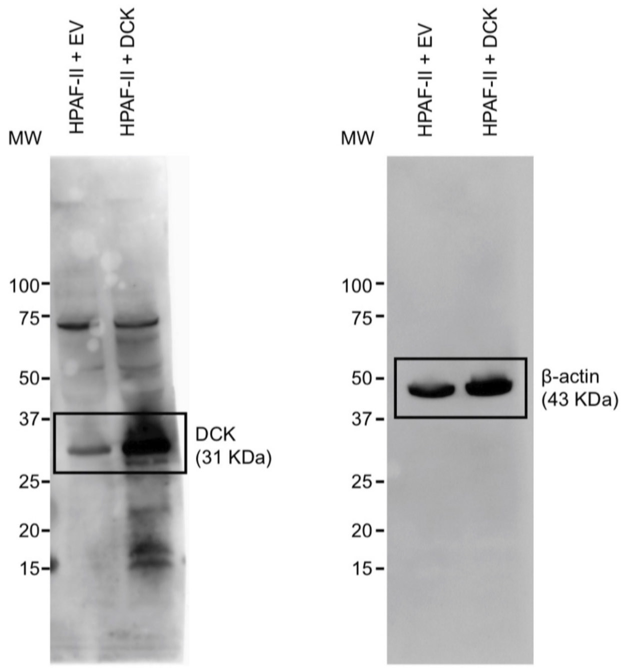

Supplementary Table 1

sgRNA oligo sequences

sgRNA	Strand	Oligo sequence
sgDCK#10	Sense	5′-CACCGAATGCATGAATGAGACAGAG-3′
	Antisense	5′-AAACCTCTGTCTCATTCATGCATTC-3′
hsgNT1	Target sequence	GCGGGCAGAACGACCCTGAC
sgGFP	Target sequence	GCACTACCAGAGCTAACTCA

Supplementary Table 2

Primer sequences used for RT-qPCR.

Gene name	Strand	Sequences
NDUFAB1	Forward	5′-TATGACAAGATTGACCCAGAGAAGC-3′
	Reverse	5′-CGTCTTCCATGGCCATGATAATCTC-3′
SDHC	Forward	5′-TGGCACTGGTATTGCTTTGA-3′
	Reverse	5′-GACTCAAAGTTCCCAGGGAGT-3′
UQCRC1	Forward	5′-TGGCTTTCAAGGGAACAAAG-3′
	Reverse	5′-CCACAGCTTTCGGCAGAT-3′
COX6C	Forward	5′-CAGCTTTGTATAAGTTTCGTGTGG-3′
	Reverse	5′-ACCAGCCTTCCTCATCTCCT-3′
ATP5PB	Forward	5′-CCTTCCTAGGTCCAGGGGTA-3′
	Reverse	5′-GGTACAGGGACAAGGTGTGG-3′
mtDNA tRNA $^{Leu(UUR)}$	Forward	5′-CACCCAAGAACAGGGTTTGT-3′
	Reverse	5′-TGGCCATGGGTATGTTGTTA-3′
B2M or nDNA β 2- microglobulin	Forward	5′-TGCTGTCTCCATGTTTGATGTATCT-3′
	Reverse	5′-TCTCTGCTCCCCACCTCTAAGT-3′
mtDNA 16S rRNA	Forward	5′-GCCTTCCCCCGTAAATGATA-3′
	Reverse	5′-TTATGCGATTACCGGGCTCT-3′
SOD2 or MnSOD	Forward	5′-GTGGAGAACCCAAAGGGGAGTT-3′
	Reverse	5′-GTGGAATAAGGCCTGTTGTTCCTT-3′
SOD1	Forward	5′-CTGAAGGCCTGCATGGATTC-3′
	Reverse	5′-CCAAGTCTCCAACATGCCTCTC-3′
BCL2	Forward	5′-GACTTCGCCGAGATGTCCAG-3′
	Reverse	5′-CAGGTGCCGGTTCAGGTACT-3′
MFN1	Forward	5′-TGGCTAAGAAGGCGATTACTGC-3′
	Reverse	5′-TCTCCGAGATAGCACCTCACC-3′
MFN2	Forward	5′-CTCTCGATGCAACTCTATCGTC-3′
	Reverse	5′-TCCTGTACGTGTCTTCAAGGAA-3′
OPA1	Forward	5′-TGTGAGGTCTGCCAGTCTTTA-3′
	Reverse	5′-TGTCCTTAATTGGGGTCGTTG-3′
DNM1L	Forward	5′-CTGCCTCAAATCGTCGTAGTG-3′
	Reverse	5′-GAGGTCTCCGGGTGACAATTC-3′
GUSB	Forward	5′-CGCCCTGCCTATCTGTATTC-3′
	Reverse	5′-TCCCCACAGGGAGTGTGTAG-3′
